# Towards sensory substitution and augmentation: Mapping visual distance to audio and tactile frequency

**DOI:** 10.1371/journal.pone.0299213

**Published:** 2024-03-26

**Authors:** Pingping Jiang, Christopher Kent, Jonathan Rossiter

**Affiliations:** 1 Department of Engineering Mathematics, University of Bristol, Bristol, United Kingdom; 2 SoftLab, Bristol Robotics Laboratory, Bristol, United Kingdom; 3 School of Psychological Science, University of Bristol, Bristol, United Kingdom; Institute of Psychology Chinese Academy of Sciences, CHINA

## Abstract

Multimodal perception is the predominant means by which individuals experience and interact with the world. However, sensory dysfunction or loss can significantly impede this process. In such cases, cross-modality research offers valuable insight into how we can compensate for these sensory deficits through sensory substitution. Although sight and hearing are both used to estimate the distance to an object (e.g., by visual size and sound volume) and the perception of distance is an important element in navigation and guidance, it is not widely studied in cross-modal research. We investigate the relationship between audio and vibrotactile frequencies (in the ranges 47–2,764 Hz and 10–99 Hz, respectively) and distances uniformly distributed in the range 1–12 m. In our experiments participants mapped the distance (represented by an image of a model at that distance) to a frequency via adjusting a virtual tuning knob. The results revealed that the majority (more than 76%) of participants demonstrated a strong negative monotonic relationship between frequency and distance, across both vibrotactile (represented by a natural log function) and auditory domains (represented by an exponential function). However, a subgroup of participants showed the opposite positive linear relationship between frequency and distance. The strong cross-modal sensory correlation could contribute to the development of assistive robotic technologies and devices to augment human perception. This work provides the fundamental foundation for future assisted HRI applications where a mapping between distance and frequency is needed, for example for people with vision or hearing loss, drivers with loss of focus or response delay, doctors undertaking teleoperation surgery, and users in augmented reality (AR) or virtual reality (VR) environments.

## Introduction

### Perception of the world

We perceive the world as a coherent and unified whole, despite receiving information from multiple different senses including hearing, touch, vision, taste, and olfaction. Information from separate sensory modalities is typically correlated, such that the different senses provide complementary and converging evidence for a meaningful perceptual experience [1]. For example, during the driving process the driver’s eyes are stimulated by the changing of distance (e.g., the speed with which road markings loom), ears are stimulated by the sound of the engine and the tyres on the road, and touch is stimulated by car and road vibrations, giving a coherent sense of the speed at which the car is moving. Because of the inherent correlation of information provided by the senses, multimodal sensory integration typically results in enhanced performance when compared to information from a single modality [2]. However, we may occasionally need to rely on one sense more heavily (e.g., waiting for traffic lights to change) or replace one sense with a different one (e.g., a reversing sensor provides a beep as guidance to the reversing driver). Furthermore, the involvement of numerous sensory modalities may give rise to cognitive strain, and information conflict between sensory modalities, for instance, as [3] suggested, using both auditory and visual channels increases the workload for users. Therefore, it is important to understand the interactions between sensory modalities, and how and when one modality may ‘support’ or ‘hinder’ the use of another.

Multi-sensory perception has been the focus of substantial investigation [4, 5], however, there is a lack of research on establishing the exact relationships between sensory modalities that present the projection from one modality feature to another modality feature, especially the mapping from visual distance into auditory and vibrotactile domains. Although it is crucial to comprehend the underlying mechanisms relating information from one modality to another, which can facilitate natural sensory projection, there have been limited endeavors to utilize sensory substitution in practical settings. Thus in this article, we focus on the commonalities of information representation across the senses, i.e., cross-modality matching [6] or sensory projection—the translation of one sense into another—that can enable one modality to be used to interpret or represent another.

### Vision and hearing loss

Among the senses, the temporary or permanent loss of vision or hearing is common and can have a significant impact on behaviour, and quality of life, both in terms of wellbeing and financial stability [7, 8], and is exacerbated for those with multiple sensory impairments [9]. The latest report from the World Health Organization [10] reveals that more than 20% of the global population is affected by hearing loss and 5.5% of the population suffers from moderate or severe hearing loss. It is estimated that more than 314 million individuals worldwide have a visual impairment and around 45 million are classified as blind [11].

There are two common solutions to aid sensory losses. First, enhancing the remaining senses through the use of tools (such as hearing aids or eyeglasses) and medical or surgical interventions. Second, incorporating alternative modalities to supplement the remaining perception. A wide range of tools and techniques have been developed to mitigate sensory loss by introducing additional sensory modes. For instance, canes have been used for many years to help individuals with visual impairments to navigate their environment. Similarly, Braille has been developed as a tactile substitute for vision-based reading and writing. More recently, technological solutions have been used to provide sensory assistance [12–17]. For example, Slade et al. [17] presented an augmented cane for people with impaired vision by integrating a set of sensors including a camera capturing environmental information, IMU (inertial measurement unit) measuring posture, LIDAR (light detection and ranging) providing distance information, and a vibration motor to impart users with haptic cues. In light of the crucial role that vision and hearing play in our daily life, our studies focus on these sensory modalities. Numerous research efforts are dedicated to providing navigation solutions for individuals with visual impairments, including technologies [16, 18] such as cameras, accelerometers, gyroscopes, and GPS systems to collect environmental information. Typically, this information is communicated through contextual audio feedback [19–21] for navigation guidance or through vibrations [22–24] for orientation instructions and obstacle warnings. Some approaches combine both audio and tactile feedback to provide a comprehensive navigation experience [25]. However, the associations between different modalities are less investigated, which may hinder people from perceiving the environment intuitively and meaningfully. Commère and Rouat [26] designed a system for encoding depth (within a range of 0–1m) with sound. In their research, five different sonifications of depth without visual feedback (including loudness, reverberation, frequency, the repetition rate of beeps, and the signal-to-noise ratio of a pure tone mixed with white noise) were compared and evaluated, and according to their results, the repetition rate of beeps is the best to encode depth while the frequency of sound provided good accuracy.

According to Tsiros [27], human-machine interfaces utilizing cross-sensory mappings, taking into account users’ perceptual knowledge, may achieve greater effectiveness in efficiently interpreting and interacting with information. Stiles et al. [28] suggested that sensory substitution can be efficiently implemented using stimuli tied to intrinsic cross-modal mappings. Hence, integrating cross-modal research into sensory substitution devices (SSDs) presents a promising avenue for enhancing natural and intuitive human-machine interactions.

### Cross-modal research with vision, sound and haptics

Cross-modality research has a long history in psychological research, with cross-modal matching often used [6]. However, the majority of research has focused on investigating the relationship between auditory and visual modalities. A typical cross-modality matching task might, for example, involve an observer adjusting the brightness of a light to indicate the loudness of a tone. More specifically, Marks [29] explored the similarities and differences in the perceptual structures of pitch, loudness, and brightness, suggesting that pitch and brightness show a strong correlation. In another example, Chiou and Rich [30] examined the cross-modal correspondence between pitch and spatial location, investigating how the brain integrates auditory and visual information to facilitate attentional orienting, and according to their findings, high-pitch sounds are associated with upward spatial locations and low-pitch sounds with downward spatial locations. Similarly, visual-auditory mappings between auditory pitch and visual sharpness were studied by [31], finding that spikier shapes were correlated with higher auditory pitch. In separate studies, higher-pitched sounds have been associated with lighter colors [32], higher luminance [33] and smaller sizes [34, 35].

Auditory pitch has been widely studied in cross-modal correspondence research, in particular, and of great relevance for substitution, is the study of congruence effects (where complementary information from two or more senses provide benefits for perception). Examples include cross-modal correspondence between pitch and visual vertical position [36], pitch and direction [37], and the mapping between pitch and object size [38]. Evans and Treisman [39], explored the spontaneous mappings of pitch to the visual features of vertical location, size, and spatial frequency and contrast, finding that the congruence effect was significant between pitch and vertical position. [40] showed that sound frequency could influence consumer responses to color lightness, from which higher frequency sounds increase attention towards a light color object, while lower frequency sounds presented the opposite phenomenon. Similarly, Sunaga et al. [41] proposed that lightness-location congruency influenced consumers’ purchases significantly. According to [39], congruent correspondences can facilitate perceptual performance. In related work [42, 43], both sighted people and blind people showed a preference for high tonal pitch with high position.

Despite the historic focus on visual and auditory modalities within psychological studies, the haptic modality has recently become a major subject of interest, extending across many fields including medical surgery, AR, VR, gaming and teleoperation [44]. There is growing interest in cross-modal sensory correlation and projection between sound and touch, as reviewed in [45, 46] as vibrotactile perception provides a pathway for people to feel the world.

The above visual-auditory and visual-tactile cross-modal matching studies collectively indicate that the correlation between vision and the two senses of hearing and touch is widespread. It is noteworthy that there has been a paucity of research conducted on visual distance and vibrotactile frequency matching, and visual distance and audio pitch matching. Exceptions include studies of navigation using sensory substitutions [47, 48] in which distance information was translated into vibration and audio frequency cues to provide real-time feedback to participants navigating through virtual mazes. This involved the prescribed conversion of distance data from detected objects into DC voltage signals, which were further transformed into sound frequencies or vibration amplitudes and frequencies. Consequently, objects closer to the user were represented by higher auditory frequencies and stronger tactile vibrations, offering instantaneous cues to facilitate navigation. However, one notable limitation is the lack of consideration and measurement of specific cross-modal projection relationships, where accurate projection and perception of distance in another modality are essential for optimizing the effectiveness of sensory substitution techniques. Another critical aspect overlooked in previous studies is the evaluation of individual perception differences in alternate sensory modalities; the neglect of individual differences may hinder the efficacy and generalizability of sensory substitution technologies.

### Aims of our study

In this work, we aim to reveal the correlation between visual distance and frequency in both auditory and vibrotactile domains. It is therefore important to consider the impact of proxemics in explaining this association. Proxemics is a field of study that explores how individuals unconsciously organize the space surrounding them, and this organization varies across different cultures [49]. As stated by [50], proxemics facilitates the identification and association of social distances with particular levels of comfort and the nonverbal communication of intentions. Hall [51] categorized social distances or “personal space” into four distinct proxemic zones: public (greater than 12 ft); social (ranging from 4–12 ft); personal (ranging from 1.5–4 ft); and intimate (ranging from 0–1.5 ft), providing a framework for understanding the various levels of physical distance that are appropriate for different types of human interactions. In particular, haptics was long considered to be intrinsically tied to proxemics [52]. A key aspect of touch is its power to express and heighten physical closeness, making it a vital element in haptic communication for interpersonal relationships that involve how individuals perceive and react to the physical proximity of others [53]. Besides, human attention tends to prioritize animate beings (such as humans and animals) over inanimate items [54], similarly, as reported by Sanz et al. [55], in their virtual proxemics experiment, participants reacted to human and inanimate obstacles differently. Therefore, it is important to consider the potential differences in mapping distance of other people and inanimate objects to frequency. We therefore use both human actors and inanimate objects in our experiments to allow a direct comparison.

To investigate the cross-modal matching relationship between visual distance, auditory pitch and vibrotactile frequency, and explore the impact of visual stimulus types (human and inanimate objects), we conducted four experiments in which we explored the perceived distance in visual-auditory and visual-tactile experiments. All tasks involved participants viewing stimuli (human or object) photographed at various distances and adjusting the frequency (auditory tone or tactile vibration) to match the perceived distance on screen.

- Experiment 1a investigated the mapping between *visual distance* and *audio frequency* using images of **humans** as the distance stimuli.
- Experiment 1b investigated the mapping between *visual distance* and *audio frequency* using images of **inanimate objects** as the distance stimuli.
- Experiment 2a investigated the mapping between *visual distance* and *vibrotactile frequency* using images of **humans** as the distance stimuli.
- Experiment 2b investigated the mapping between *visual distance* and *vibrotactile frequency* using images of **inanimate objects** as the distance stimuli.

## Materials and methods

### Participants

The experiments ran from October 2021 to April 2022. Participants (aged 18–36) were from the University of Bristol and participated in return for course credit or a small financial reimbursement. All participants self-reported normal or corrected-to-normal vision and hearing. We required participants to have use of both hands for the tactile experiments, with the dominant hand used for mouse control and the non-dominant hand for tactile stimulation. There were 26 participants for Experiment 1a, 23 for 1b, 27 for 2a, and 25 for 2b. Before engaging in the research activities, all participants gave informed consent. The process of obtaining consent was conducted using our online experiment webpages, where participants were provided with a digital participant information sheet and a digital consent form before starting the experiment. Throughout the duration of the experiment, all user data were fully anonymized, ensuring that no personal information was gathered or retained. This study was approved by the Faculty of Engineering Research Ethics Committee at the University of Bristol (study reference number: 9402). The sample size was based on a minimum sample of 20 participants and a maximum of 30 within a reasonable time frame.

### Procedure

**Fig 1:**
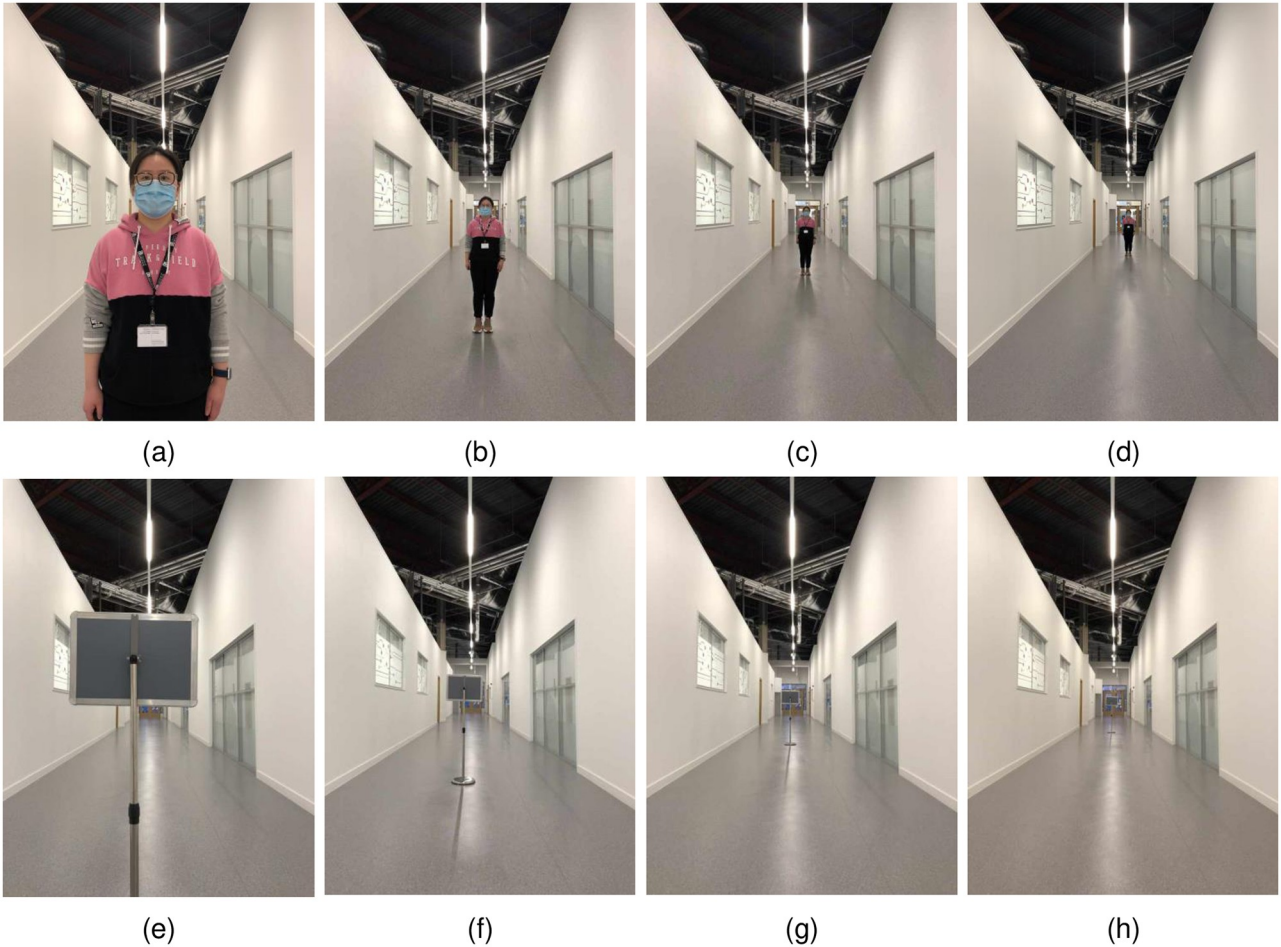
Two image stimuli at different positions. (a) Human standing at 1m distance from the camera, (b) Human at 4m, (c) Human at 8m, (d) Human at 12m, (e) Signpost object at 1m distance from the camera, (f) Signpost at 4m, (g) Signpost at 8m, (h) Signpost at 12m.

In all experiments, we manipulated the distance the item appeared away from the camera viewpoint (in the physical world this was between 1 and 12 meters, in equal increments of 0.5m) referred to henceforth simply as ‘distance’. In Experiments 1a and 2a, photographs of three humans were used as visual stimuli, and in Experiments 1b and 2b photographs of three inanimate objects (a coat rack stand, a chair, and a signpost (as shown in Fig 1e)) were used. The dependent variable in Experiments 1a and 1b was the audio frequency chosen to match the distances, and in Experiments 2a and 2b, the tactile vibration frequency chosen to match the distances.

Stimuli were created by taking photos that presented distance information in a simple and bright environment (a corridor) which enabled users to focus on the object position relative to the camera lens position (aperture: f/1.8; focus length: 3.99mm; height from the ground: 1.4m). We took photographs of three humans and three inanimate objects at each of the 23 different distances, totaling 138 images. All objects (human and inanimate) were located in the middle of the corridor, and the camera was fixed in position such that the reference environment would be the same in each image. After taking these photos, simple image processing was applied to remove the shadows of the objects on the corridor windows and to erase the position markers on the ground. As the experiment took place during the COVID-19 pandemic, all humans were wearing face coverings.

Participants completed Experiments 1a and 1b (auditory matching) online using their own computers for the sake of the safety of participants due to the COVID-19 pandemic. Participants were instructed to perform the experiment in a quiet environment without any interruption. The auditory tones (Experiments 1a and 1b) were sine waves played through the participants’ own audio devices (speakers or headphones). In the experiment, the auditory frequencies ranged from 47 Hz to 2,764 Hz. The pitch (frequency) was controlled by the experiment but the loudness (intensity) was controlled by participants and was set as a constant throughout the experiments. Participants were instructed to adjust the volume, controlling the intensity, before commencing the experiment. The hearing range for a healthy young individual is approximately 20 Hz to 20 kHz, thus, we chose this frequency range for our experiment [56]. But higher-frequency sounds, especially those exceeding 2,000 Hz, can potentially be uncomfortable or even harmful under prolonged exposure [57]. Given our 30-minute experiment duration, we initiated the frequency range from a low-frequency sound (47 Hz) to a moderately high frequency (2,764 Hz). This limited range also ensured participants were able to utilize the whole range without taking too long in dialing through the minimum to maximum frequencies and was within the reproduction range of the diverse audio devices used by the remote participants. Participants were able to set their volume levels so that all tones were comfortable.

**Fig 2:**
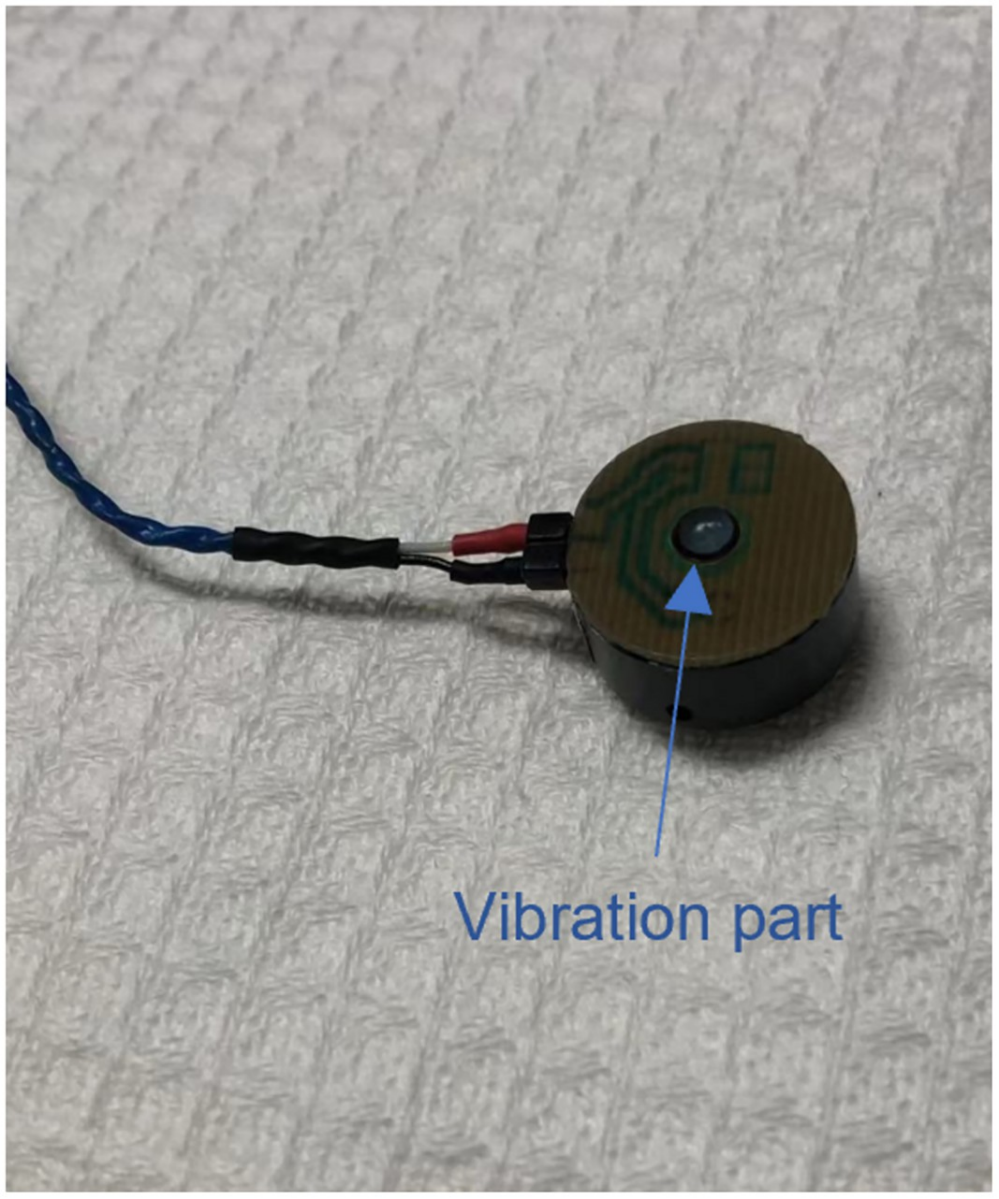
Tactor used in the experiment. Tactor dimension: 18 mm diameter and 12 mm height.

For Experiment 2a and 2b (vibrotactile mapping), participants completed the experiment while sitting in front of a 21” LCD monitor running at 1,280 × 1,024 resolution at 60 Hz at approximately 50 cm viewing distance in a quiet lab. Vibrotactile stimuli were played through a vibrotactile electromagnetic solenoid-type stimulator (a Tactor made by Dancer Design, as shown in Fig 2) driven through a JUNTEK DPA-1698 amplifier and attached to the index finger of participant’s non-dominant hand. The minimum vibration frequency was 10 Hz, and the maximum vibration frequency was 99 Hz. This range was used to ensure a relatively constant force on the fingertip and that participants could sufficiently feel the difference (in terms of just noticeable differences) within the range of vibrations. During the experiments, the participants were asked to rest their hand on a table (fingertip down) while their hand was wrapped in a towel to dampen any vibration sound. Participants also wore noise-canceling over-ear headphones throughout the experiment.

**Fig 3:**
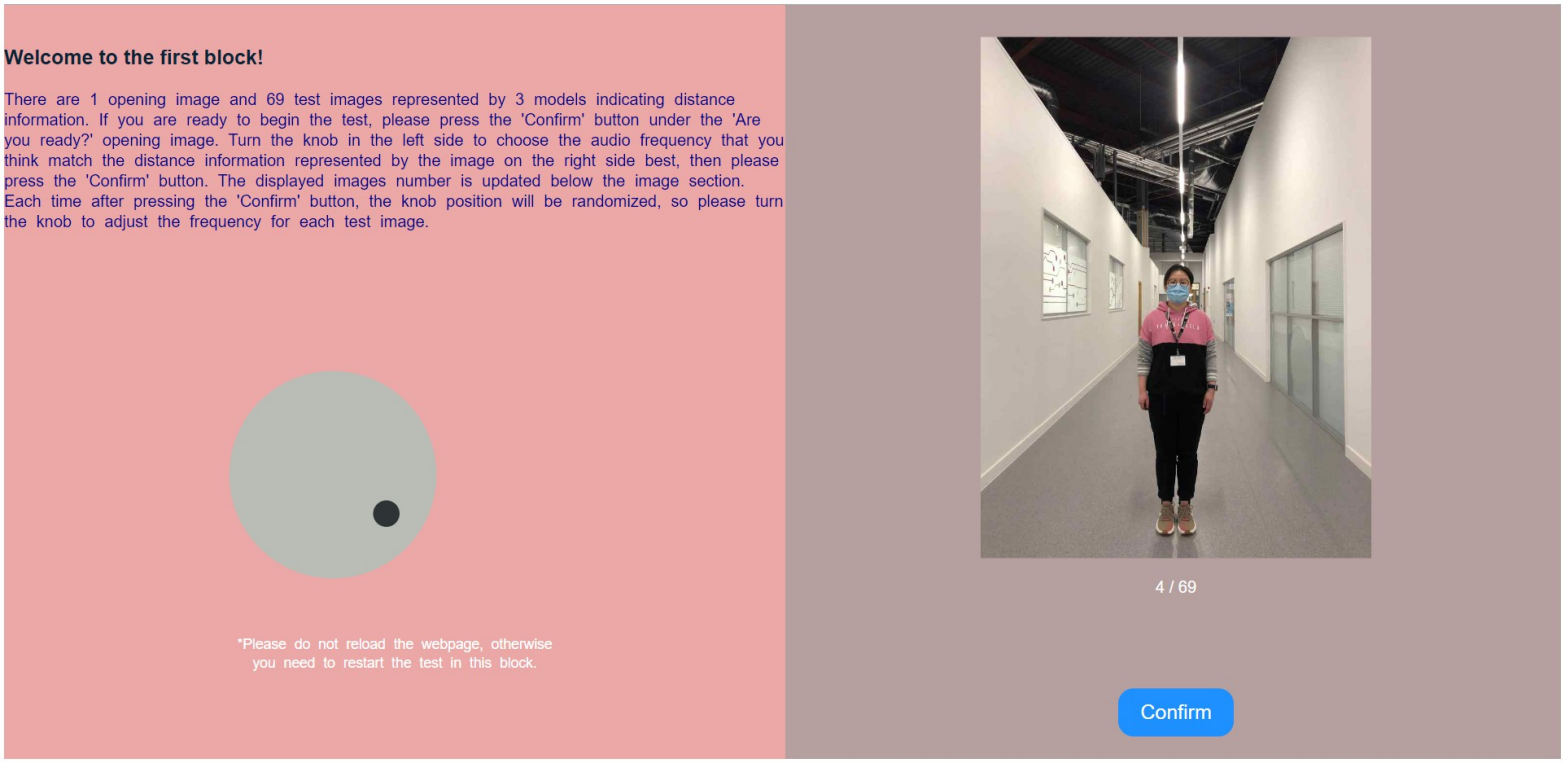
Experiment webpage of the matching test. This webpage shows image stimuli on the right and a user-adjustable dial on the left to select a matching audio or tactile vibration frequency when users were given randomly displayed image stimuli.

A series of custom-written webpages (see example in Fig 3) were used to run all experiments. Participants were provided with the web address of the experiment and after clicking the URL link, participants were taken to the online participant information sheet as the landing page. Following the information sheet, participants were presented with a preview of the visual stimuli. This preview consisted of a sequence of nine images displayed sequentially, including all three humans placed at the furthest, nearest, and intermediate distances. Thus the presentation of the initial visual stimuli allowed participants to acquire a comprehensive understanding of the range of distances involved in the task. Subsequently, participants were directed to another preview webpage, where they were given the opportunity to practice utilizing the response dial while simultaneously hearing or feeling the frequency that had been selected. Participants used a mouse to turn a virtual dial displayed onscreen to input their responses. To increase frequency, participants held the left mouse button while turning the dial in a clockwise direction, whereas turning the dial counter-clockwise decreased the frequency. During the study, participants were allowed to continually adjust the dial until they felt the frequency best matched the visual distance, with no time limit. The dial could not be rotated counter-clockwise past the minimum frequency or clockwise past the maximum frequency. Additionally, in the auditory experiments only, the frequency step is non-linear due to the inability to detect small frequency changes (for frequencies less than 4,000 Hz, the smallest detectable change is approximately 0.2–0.3% [58]), and to encourage participant to use the full range of frequencies (and not to ‘give up’ if dialing took too long). Consequently, our frequency step for auditory frequency spans from 1 Hz to 6 Hz nonlinearly, for example, 1 Hz frequency step for the frequency range of [47 Hz,500 Hz], and 2 Hz frequency step for the frequency range of [501 Hz,1000 Hz], and so on. As reported in [59], the frequency discrimination for fingertip vibration is nonlinear. Specifically, for vibration on the index fingertip, the discrimination frequency is approximately 7 Hz at 20 Hz vibration frequency, around 10 Hz at 50 Hz vibration frequency, and approximately 21 Hz at 100 Hz vibration frequency. In our vibrotactile experiments, we employed a 1 Hz vibration frequency step, ensuring that participants could accurately detect various vibration frequencies within the specified range.

After participants were familiar with the task, they completed a consent form and then started the experimental trials. Each participant engaged in the experiment by completing three identical blocks. Each block comprised 69 trials, featuring images of either 3 human models or 3 object models at 23 different distance positions. These images were presented in a random order to ensure variability in the experimental conditions. In each trial of the experiment, the visual stimulus was shown on the right of the webpage with a size of 600px × 450px and the response dial on the left. The dial was reset to a random frequency at the beginning of each trial, thereby ensuring that participants did not anchor their responses to any part of the frequency range. The auditory or tactile stimuli were started only when the participant started moving the dial. Once the participant had selected the desired response frequency using the dial, they clicked on the ‘Confirm button’ (see Fig 3) and the next image was presented. At the end of a block, participants were allowed a short break before completing the next block. Overall, the experiment (207 trials) lasted approximately 30 mins.

## Results

**Fig 4:**
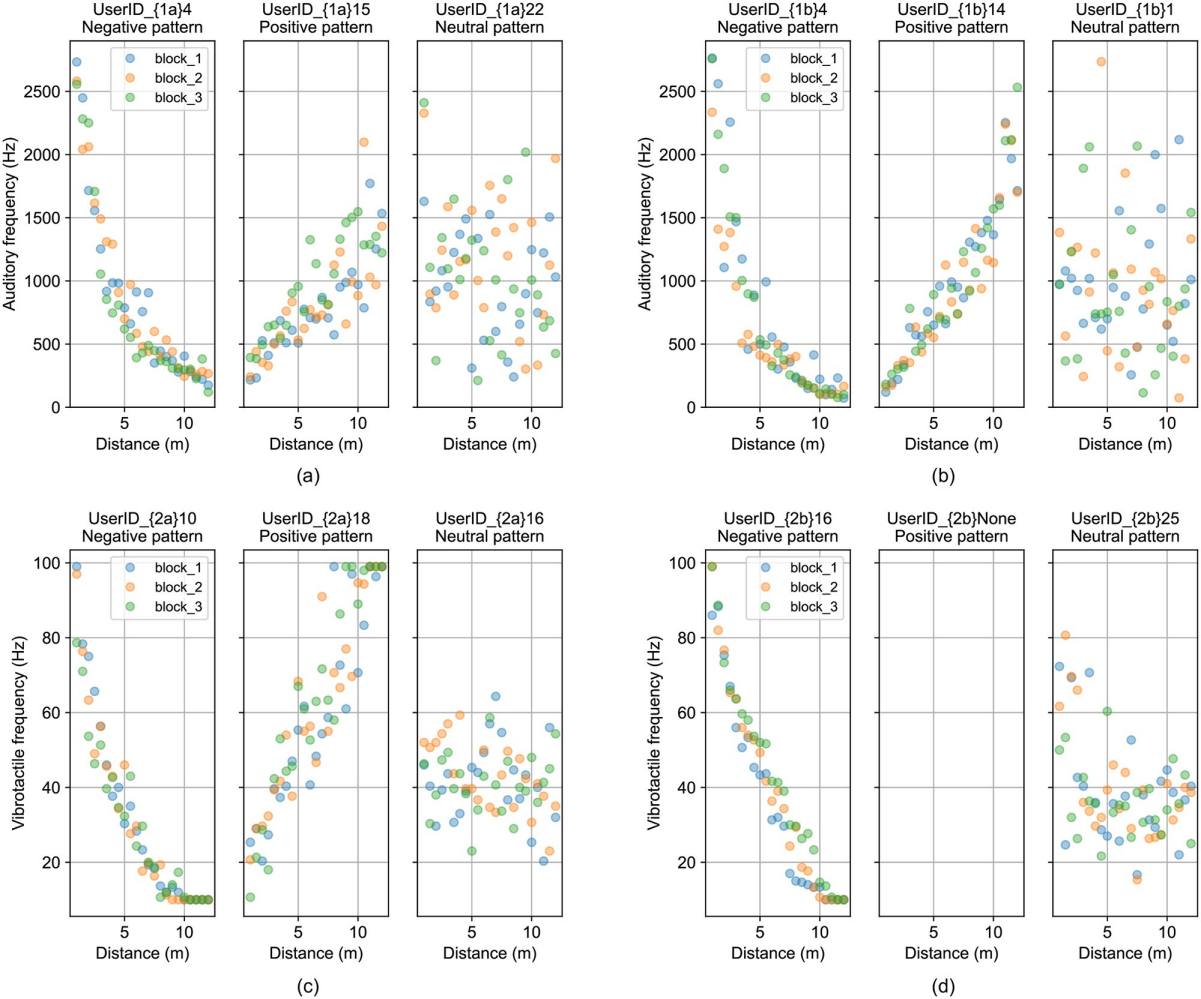
Auditory or vibrotactile frequencies as a function of perceived distance. (a) Experiment 1a, three patterns: negative, positive, and neutral. (b) Experiment 1b, three patterns: negative, positive, and neutral. (c) Experiment 2a, three patterns: negative, positive, and neutral. (d) Experiment 2b, two patterns: negative and neutral.

First, for each experiment, we plotted matched frequency as a function of distance across the three blocks. Randomly chosen representative participants from each experiment were shown in Fig 4. An initial inspection of the data showed that while most participants demonstrated a negative relationship between distance and frequency (further distances were associated with lower frequencies), not all participants followed this pattern, with some showing the reverse, positive, relationship (with further distances associated with higher frequencies) and others showing an apparently random response pattern.

**Fig 5:**
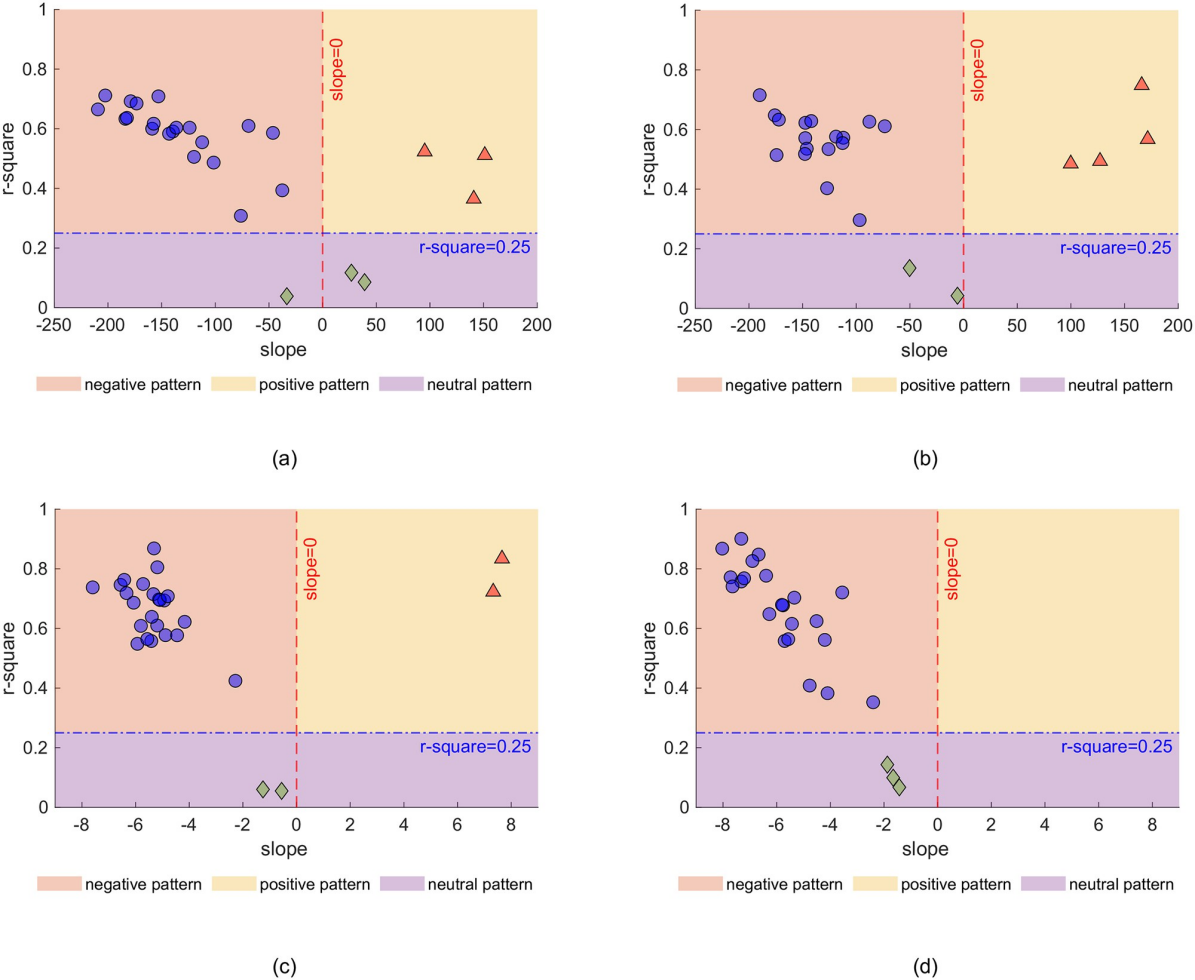
Data partitioning into three types. For Experiment (a) 1a, (b) 1b, (c) 2a, (d) 2b respectively, applying linear fit parameters (*R*^2^ and slope *b*) to partition the user data into negative pattern, positive pattern and neutral pattern. One dot represents a single participant.

Next, to classify the relationship between frequency and distance for each participant, we fit a simple linear model (of the form *y* = b*x* + *a*) to each participant’s data. We then partitioned the data into three types using two criteria: the slope value (*b*, positive, negative, or flat) and *R*^2^) goodness of fit of the model to the data. We set the *R*^2^ threshold value as 0.25, and compared the *R*^2^ for each participant to this value to decide whether the participant was responding in a consistent manner, or was employing a strategy that appeared random (note, this would also capture a strategy whereby participants just guessed without engaging in the task). As shown in Fig 5, if the *R*^2^ of a user was greater than the threshold and the slope was smaller than 0, then the user’s data were classified as representing a negative relationship between distance values and frequencies. If the *R*^2^ of a user was greater than the threshold and the slope was greater than 0, then this user’s data were classified as representing a positive relationship between distance and frequency. Finally, if the *R*^2^ of a user was smaller than the threshold, then it was defined as randomly scattered and discarded from further analysis.

Fig 5a shows the three relationships for Experiment 1a: 77.0% showed a negative relationship, 11.5% showed a positive relationship, and 11.5% showed no relationship. In Experiment 1b (see Fig 5b) 74.0% of participants showed a negative relationship, 17.4% a positive relationship and 8.6% participants showed no relationship. In Experiment 2a (Fig 5c) 85.2% showed a negative relationship, 7.4% a positive relationship and 7.4% no relationship. In Experiment 2b (Fig 5d) 88% showed a negative relationship and 12% a neutral relationship.

Second, we examined the effects of both block and image type (human or inanimate object) on chosen frequency as a function of distance. To do this we calculated the slope value of each block or image type for each participant. We entered the slope value into a repeated measures ANOVA for human models and object models respectively. Across all experiments, there was no significant difference due to block, for Experiments 1a (*F*(2, 50) = 1.589, *p* = 0.214), Experiment 1b (*F*(2, 44) = 1.329, *p* = 0.275), Experiment 2a (*F*(2, 52) = 0.994, *p* = 0.377) and Experiment 2b (*F*(2, 48) = 0.128, *p* = 0.880). Similarly, different image models did not affect perception in each experiment: Experiment 1a (*F*(1.272, 31.809) = 0.749, *p* = 0.424), Experiment 1b (*F*(2, 44) = 2.637, *p* = .083), Experiment 2a (*F*(2, 52) = 2.820, *p* = 0.069) and Experiment 2b (*F*(2, 48) = 2.916, *p* = 0.064). Because neither the block factor nor the image type factor had a significant impact on the data, in the main analysis we averaged over block and image.

Next, we investigate the difference between animate visual stimuli and inanimate visual stimuli as [54, 55] highlighted a difference in perception between humans and objects. We compared the slopes of participant responses in Experiment 1a with those of participants in Experiment 1b, and likewise compared Experiment 2a with 2b. We calculated a total of 23 pairs of distance and auditory frequency data for each participant, and a simple linear slope was fit to each participant’s data set. This resulted in the computation of 49 slope values for visual-auditory experiments (26 from Experiment 1a and 23 from 1b) and 52 slope values for visual-tactile experiments (27 from Experiment 2a and 25 from 2b). An independent samples t-test on the slope values revealed no significant difference between the slope of the relationship between distance and frequency for human images compared with object images for visual-auditory matching (*t*(47) = −0.334, *p* = 0.740 > 0.05), or for vision-tactile matching (*t*(50) = 1.514, *p* = 0.136 > 0.05). Therefore, we concluded that there is no significant difference between the perception of distance for human images compared with inanimate images in this study.

Finally, we aimed to further quantify the shape of the relationship between distance and frequency for the two distinctive groups showing a consistent pattern of response (either a positive or negative relationship). We fit a selection of characteristic curves (linear, exponential, power and natural logarithmic) to the data from each group and compared the *R*^2^ values to determine the best-fitting function for each set of data.

**Fig 6:**
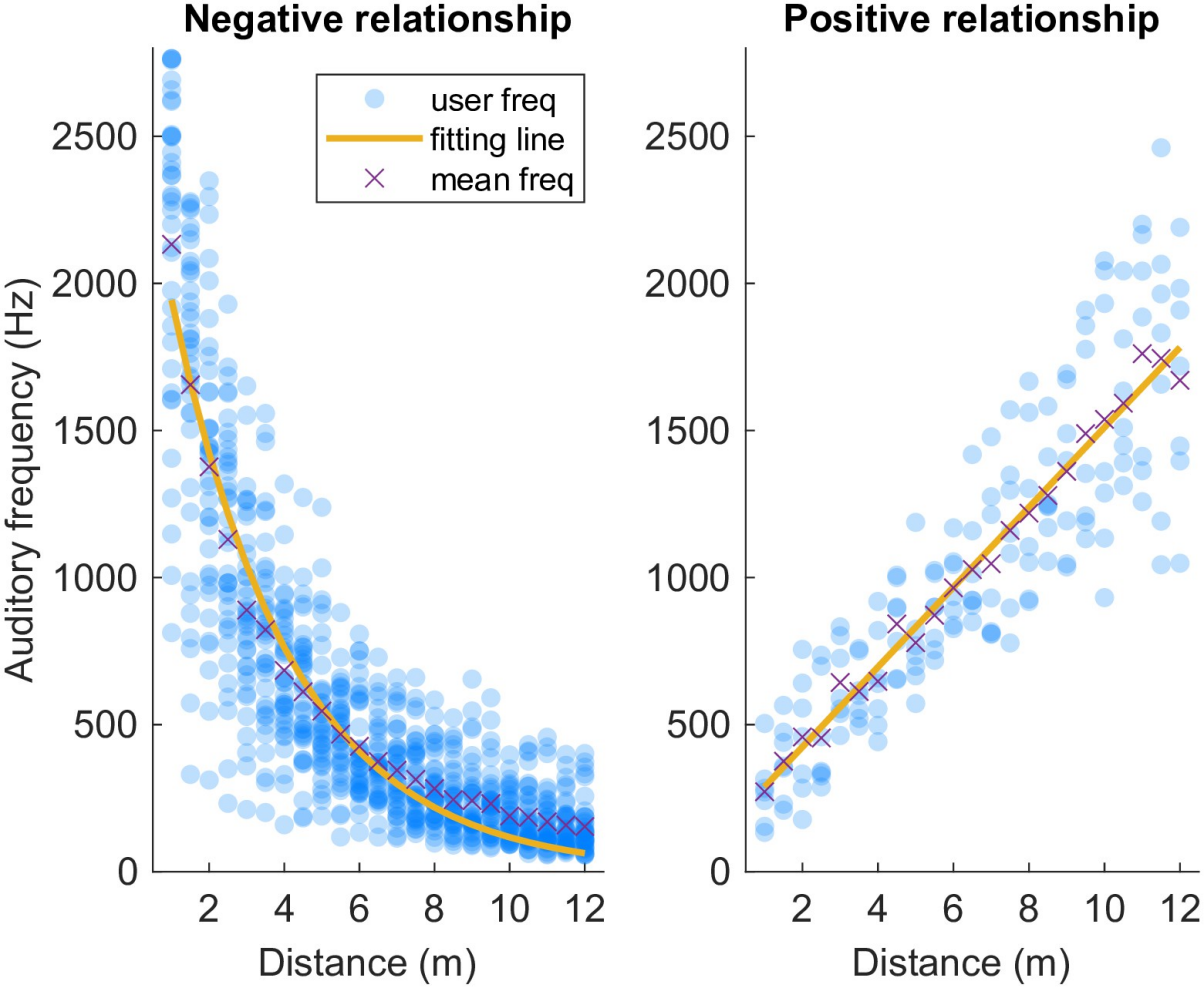
Vision-to-audio experiment fitting function plot. For Experiment 1a & 1b, a negative pattern (Eq 1a) and a positive pattern (Eq 1b) functions were generalized across averaged individual participant data to represent the relationship between auditory frequency and visual distance.

For vision-to-audio experiments, the *R*^2^ mean goodness of fit values for a negative relationship between distance and frequency were: natural logarithmic (*R*^2^ = 0.7703), power (*R*^2^ = 0.7852), exponential (*R*^2^ = 0.7858) and linear (*R*^2^ = 0.6067). Fig 6) shows the best fitting exponential function of the average data, as given in Eq 1a. For the positive relationship between distance and frequency in the vision-to-audio task, the *R*^2^ values were: natural logarithmic (*R*^2^ = 0.6870), power (*R*^2^ = 0.7610), exponential (*R*^2^ = 0.7400) and linear (*R*^2^ = 0.7637). The linear function fitted the data well, and the corresponding function and plot are demonstrated in Eq 1b and Fig 6 respectively.

$$\begin{array}{c} y_{negative}=2655.43e^{-0.31x} \end{array}$$ (1a)

$$\begin{array}{c} y_{positive}=152.46+135.82x \end{array}$$ (1b)

**Fig 7:**
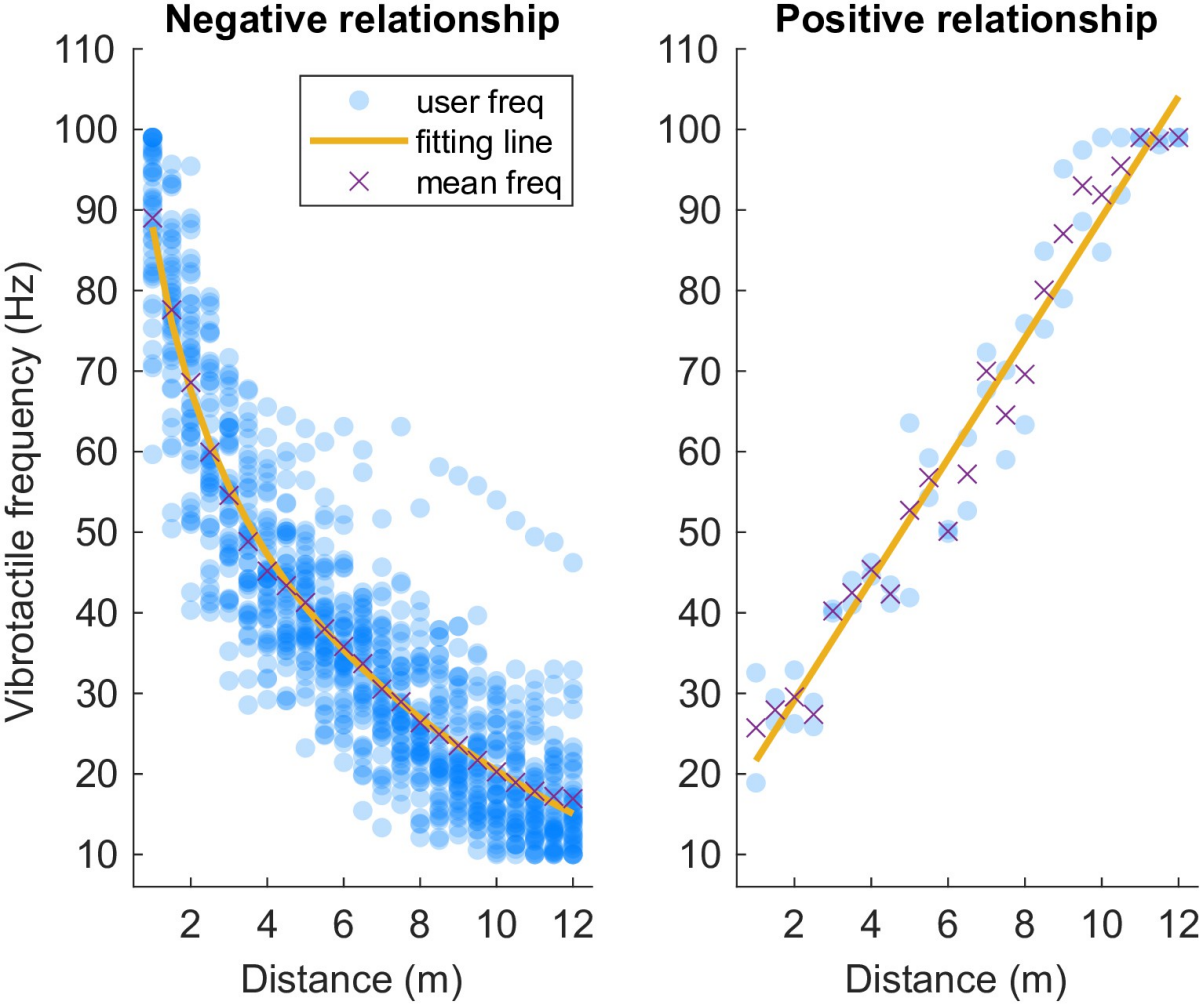
Vision-to-vibrotactile experiment fitting function plot. For Experiment 2a & 2b, a negative pattern (Eq 2a) and a positive pattern (Eq 2b) functions were generalized to represent the relationship between vibrotactile frequency and visual distance.

For the vibrotactile tasks, the best fitting functions for the negative relationship were: natural logarithmic (*R*^2^ = 0.8622), power (*R*^2^ = 0.8303), exponential (*R*^2^ = 0.8477), and linear (*R*^2^ = 0.7652). According to the *R*^2^ values, the natural logarithmic curve (Fig 7) best captured the negative relationship between visual distance and tactile vibration frequency, as expressed by Eq 2a. For the participants showing a positive relationship between visual distance and tactile vibration frequency: natural logarithmic (*R*^2^ = 0.8261), power (*R*^2^ = 0.9300), exponential (*R*^2^ = 0.9213) and linear (*R*^2^ = 0.9414). Fig 7 and Eq 2b show the best-fitting linear function.

$$\begin{array}{c} y_{negative}=88.02-29.68\;\text{ln}\;x \end{array}$$ (2a)

$$\begin{array}{c} y_{positive}=14.17+7.49x \end{array}$$ (2b)

We conducted an exploratory analysis of the response time concerning these three distinct relationships. Given that half of our experiments (vision-to-audio matching experiments) are conducted online, with participants having the flexibility to manage their own time for completing the tasks, we face limitations in obtaining precise response time measurements. Thus we carried out an evaluation only for the vibrotactile experiments. Following a comparison of response times between the random pattern group and the two other groups featuring typical patterns in vision-to-vibrotactile matching experiments, our findings indicate no statistically significant difference between these groups (average trial response time of each pattern: Negative pattern (M = 7.27, SD = 2.25); Positive pattern (M = 6.17, SD = 0.71); Neutral pattern (M = 6.47, SD = 1.34). This is perhaps surprising as we expected the random groups to involve more guessing (faster response times).

## Discussion

Our study aimed to quantify the cross-modal matching relationship between visual distance and auditory frequency (Experiments 1a and 1b) and vibrotactile frequency (Experiments 2a and 2b). In the vision-to-audio experiments, around 76% of participants (by calculating the average from Experiment 1a and Experiment 1b) demonstrated a negative relationship between visual distance and auditory frequency (i.e., closer distances were associated with higher frequencies) which was well described by an exponential function, meaning that frequency increased more rapidly the nearer the object appeared to the observer. More distant objects were less well discriminated, i.e., people are less sensitive to more distant objects in that the mapping was much flatter as distance increased. A positive relationship between visual distance and auditory frequency was observed in 10% of participants and is well described by a linear function, where close distances were associated with lower frequencies. In the vibrotactile study, 87% of participants demonstrated a negative relationship between visual distance and vibrotactile frequency, which was well explained by a natural logarithmic function. Only 4% of participants demonstrated a positive relationship between visual distance and vibrotactile frequency, which was well described by a linear function. It is worth noting that around 10% of the participants showed no readily discernible relationship between distance and frequency. Based on the average response time discussed above, although we found no correlation between response time and relationship curve patterns, future studies may still be needed to ascertain possible individual differences and ensure participants are more thoroughly questioned about their strategy or approach.

Similar relationships were observed in both vision-to-audio and vision-to-vibrotactile experiments. The majority of participants exhibited a negative correlation between distance and frequency, while the significance of a positive correlation cannot be overlooked in both auditory and tactile experiments. As reported in [60], the processes of hearing and sensation involve transforming physical events into neural codes based on frequencies. This suggests a close connection between the auditory and somatosensory systems, and our study is in line with evidence supporting the anatomical linkage of neural substrates for audition and somatosensation. Additionally, other research indicates that the encoding mechanisms at the receptor surfaces for audition and vibrotactile perception are analogous [61], and physiological mechanisms underlying vibrotactile and auditory perception likely share common origins in both animals and humans [62, 63].

In this study, it is important to highlight our assumed use of a relative general mapping between modalities (bounded by the stimulus and response ranges we used), rather than a direct absolute mapping. Although our data do not directly suggest that a general processing mechanism exists for all modalities (e.g., as proposed by [64]), we do assume that humans can flexibly remap the range of one modality onto another (we saw no difference across blocks, suggesting a stable mapping was established quickly within the experimental context). In the context of our cross-modal research, cross-modal correspondences are often demonstrated by examining the interaction between pairs of stimuli in different sensory modalities, considering various characteristics [65]. It is not clear if these connections are definite or dependent on the context. In other words, we are unsure if they come from fixed values connecting the two modalities (an absolute mapping) or if they’re flexible and influenced by past instances of stimuli from different senses (relative mapping). As reported by Brunetti et al. [66], the pitch and size correspondence experiment suggests that cross-modal correspondence is relative in nature, and we support this view here, with how easily participants are able to quickly map one range of distances onto one range of frequencies. Importantly, the fact that some participants show the reverse pattern (shorter distances with low frequencies) suggests that individual differences in mapping should be considered in the design of SSDs.

We designed these vision-to-audio and vision-to-vibrotactile cross-modal experiments with the goal of contributing to the development of assistive technologies for individuals with sensory loss and providing a foundation for future sensory augmentation. Based on our results, we believe that while the relationship between distance and frequency can be approximated by simple linear and nonlinear functions, these cannot capture the complexity of the cross-modal interaction and may not be the most intuitive mapping. Although we found no distinction between human and object images, the influence of proxemics should still be further investigated, as proxemics has been found to play an important role in social communication. As stated in [67], discomfort is linked to breaches of personal space, but the specific relationship between the level of discomfort and the distance between individuals has not been defined yet and likely is dependent on contextual and individual difference factors. The dominance of a monotonic negative relationship in our visual distance mapping in both auditory pitch and vibrotactile frequency experiments might be potentially attributed to proxemics, where individuals may experience a reduction in comfort levels as others (whether human or inanimate objects) approach closer, and as a result, individuals may be more likely to respond to strong cues. Our design did not allow us to fully explore the differences between extra-personal and peri-personal space, because the shortest distance was 1 meter. Future experiments should look at whether the function relating distance to frequency changes as a direct function of extra-personal or peri-personal space, and the role that other social factors, such as threat perception, play in how the function might change. It is worth exploring other factors such as sensory acuity, cultural background and societal norms in the future to evaluate how individual differences impact on the relations drawn from our experiments. Furthermore, exploration of the underlying neurophysiology could potentially benefit our understanding of this particular cross-modal matching and how this interacts with peri-personal and extra-personal space.

These fundamental relations can be applied to assistive technologies, such as navigation devices, to provide people who are deaf or blind with precise and personalized distance information using auditory or vibrotactile cues. In addition, our findings shed light on the mechanisms of human cross-modal sensory correlation and substitution and have significant practical implications in various domains, including gaming, driving, and human-robot interaction. Specifically, our research can help in translating visual distance to auditory frequency or tactile vibration more intuitively, which might potentially be applied to enhance situational awareness, reduce response time, and improve driving performance. Research [68] shows that delays in response time resulting from glances away from the forward roadway can last for several seconds and can increase the likelihood of a crash. In such a situation, a quick auditory alert or vibrotactile warning representing the distance to the object in front could be an effective approach to reduce response time and improve driving safety [69]. Previous studies [70] have also highlighted the potential of vibrotactile cues in directing a driver’s visual attention towards relevant information in a driving scenario, leading to faster reactions and improved driving performance. Our study provides an indicator for the need of more accurate distance to frequencies mappings (which a individualised).

In AR and VR experiences, the visual system relies on depth cues to perceive distances, such as perspective, size, shading, and motion parallax [71]. These cues are generated by the virtual or augmented environment and presented to the user’s eyes. While modern AR and VR systems have made significant advancements in providing realistic depth cues, there can still be instances where human perception of distance may not align perfectly with physical reality. Our cross-modal matching function serves as a valuable tool in reducing potential disparities between perceived and physical distances within AR and VR environments. This function effectively addresses this concern by presenting auditory or vibrotactile cues that are carefully translated from visual distance cues. By incorporating these additional sensory modalities, users are provided with a more comprehensive perception of distance, thereby minimizing discrepancies that may arise. Furthermore, our cross-modal matching functions facilitate accurate perception of distance changes within AR and VR experiences. Through the integration of these functions, users can reliably discern alterations in distance, ensuring a heightened level of realism and immersion. By aligning the perceived distance changes with the corresponding physical changes, our cross-modal matching functions enable a more precise and consistent perception of spatial transformations.

Additionally, when considering the application of our investigation to SSDs, it is noted that our current approach is one-dimensional. This differs from some SSDs that convey various information through sound or touch at the same time, making them multi-dimensional. For example, some devices use musical notes to communicate color and shape information [13]. While many previous SSDs can provide multiple streams of information simultaneously, they often require participants to learn or be trained to understand the connections. In this study, our focus was on establishing an intuitive mapping between modalities, prioritizing a natural and swift connection. This emphasis on intuitiveness holds particular relevance in time-critical situations [72]. It is important to acknowledge the inherent trade-off between the complexity/fidelity of the representation and the speed/ease of interpreting the mapping. In the specific context of our application, we sought to understand the fundamental one-dimensional functions that map distance to frequency. Nevertheless, future research should explore and compare different mappings, including the transition from one dimension to multiple dimensions. This exploration aims to construct a cross-modal mapping system that is both effective and efficient in enhancing sensory perception.

In our experimental setup, we employed 2D visual images instead of 3D representations to convey distance. This choice introduces certain factors that may impact the generalizability of our results. A notable limitation associated with the utilization of 2D images for distance representation is the absence of depth perception, which poses challenges in accurately assessing the relative distances between objects [73]. In 2D images, visual cues like linear perspective, overlap, and shading are used to imply depth. These cues are tricks that create the illusion of three-dimensional space on a flat surface. In 3D environments, depth is inherent. Objects have actual spatial coordinates, and their size and position in the scene are accurately represented. Viewers can naturally perceive depth through binocular vision and the parallax effect as they move through the environment. It should be noted that, in our experiment, we sought to address this limitation by constructing a fixed environment setting that serves as a reference, providing contextual information intended to facilitate viewers’ understanding of the intended distances. Nevertheless, the implementation of 3D visualization techniques (such as stereoscopic visualization [74], VR [75], AR [76], depth maps [77] etc.) for presenting image data holds the potential to offer novel perspectives and enhance the accuracy of distance perception. By creating a three-dimensional representation, crucial depth information can be incorporated, allowing viewers to more accurately gauge distances between objects. This may prove particularly valuable when precise measurement is required.

Furthermore, there are some limitations related to the hearing requirement and device control in the vision-to-audio experiments which were carried out online due to social distancing restrictions in place from the COVID-19 pandemic. Given the diverse equipment used by participants, uncertainties exist regarding the reproduction of the entire frequency range. Additionally, we lack information about how the equipment was employed in the experiment—whether it be free-field, headphones, or earphones. Despite highlighting the importance of hearing ability during participant recruitment and at the experiment’s outset, there may still have been individuals with hearing deficits, potentially affecting their frequency and volume perception. Fortunately, our experiment benefited from recruiting young university students, reducing the likelihood of hearing loss. While any individual differences are unlikely to impact the overall results, these uncontrolled variations may have contributed to the different patterns of mapping (i.e., the positive or negative relationship between frequency and distance). For future studies, conducting these experiments in a controlled lab environment will ensure an equivalent level of control to the tactile experiments.

In order to fully apply our findings to sensory substitution, it is important that future work should expand upon our current framework by conducting additional studies that investigate the relationship between auditory/vibrotactile frequency and visual distance in reverse (i.e., present a frequency and have participants generate a distance). Our present study indicates the existence of monotonic relationships for both vision-to-audition and vision-to-vibrotactile cross-modal projections. From our experiments, an individual facing hearing challenges can utilize combinations of visual and tactile cues, and our study also yields valuable insights for establishing audio and tactile solutions customized for the needs of individuals who are blind. Future studies should analyse the relationships of audio-to-vision and vibrotactile-to-vision cross-modal matching, and compare and contrast these outcomes with our current study’s findings to explore the complete bidirectional sensory mapping relationships. Ultimately, we must scale up and evaluate these underlying correlations on navigation devices to real-world conditions, in which people will be given audio or vibrotactile cues to learn to “feel distance” if they are to become useful mappings for sensory substitution in the real world.

## Conclusion

In this study, we have successfully quantified the underlying relationship between visual distance and audio frequency, and elucidated the association between visual distance and vibrotactile frequency. Our results reveal strong monotonic relationships between visual distance and both auditory and vibrotactile frequency, with distinct positive or negative mapping functions observed in different participants. These findings have important implications for assistive human-robot interaction technologies, particularly in scenarios where individuals may experience sensory loss or dysfunction. Additionally, our study results may play an important role in mitigating potential discrepancies between perceived and physical distances in AR and VR contexts. Through the integration of auditory or vibrotactile cues derived from visual distance cues, our cross-modal matching function can enhance the overall perception of distance and ensure the accurate perception of distance changes, ultimately contributing to a more realistic and immersive user experience. Importantly, our results highlight the importance of considering individual differences in mapping distance to frequency for future applications.

## Supporting information

S1 Dataset (TXT)
